# Immunomodulatory properties of human adult and fetal multipotent mesenchymal stem cells

**DOI:** 10.1186/1423-0127-18-49

**Published:** 2011-07-18

**Authors:** Pei-Min Chen, Men-Luh Yen, Ko-Jiunn Liu, Huey-Kang Sytwu, B-Linju Yen

**Affiliations:** 1 Regenerative Medicine Research Group, Institute of Cellular and System Medicine, National Health Research Institutes (NHRI), Zhunan, Taiwan; 2 Department of Primary Care Medicine, and Department of Obstetrics/Gynecology, College of Medicine, National Taiwan University and Hospital, Taipei, Taiwan; 3 National Institute of Cancer Research, NHRI, Tainan, Taiwan; 4 Graduate Institute of Microbiology and Immunology, National Defense Medical Center, Taipei, Taiwan; 5 Department of Obstetrics/Gynecology, Cathay General Hospital Shiji, Taipei, Taiwan

**Keywords:** mesenchymal stem cells, bone marrow, fetal, multilineage differentiation, immunomodulation, T lymphocytes, natural killer lymphocytes, dendritic cells, major histocompatibility complex (MHC) molecules

## Abstract

In recent years, a large number of studies have contributed to our understanding of the immunomodulatory mechanisms used by multipotent mesenchymal stem cells (MSCs). Initially isolated from the bone marrow (BM), MSCs have been found in many tissues but the strong immunomodulatory properties are best studied in BM MSCs. The immunomodulatory effects of BM MSCs are wide, extending to T lymphocytes and dendritic cells, and are therapeutically useful for treatment of immune-related diseases including graft-versus-host disease as well as possibly autoimmune diseases. However, BM MSCs are very rare cells and require an invasive procedure for procurement. Recently, MSCs have also been found in fetal-stage embryo-proper and extra-embryonic tissues, and these human fetal MSCs (F-MSCs) have a higher proliferative profile, and are capable of multilineage differentiation as well as exert strong immunomodulatory effects. As such, these F-MSCs can be viewed as alternative sources of MSCs. We review here the current understanding of the mechanisms behind the immunomodulatory properties of BM MSCs and F-MSCs. An increase in our understanding of MSC suppressor mechanisms will offer insights for prevalent clinical use of these versatile adult stem cells in the near future.

## 1. Mesenchymal stem cells: Definition and functional capacity

Human mesenchymal stem cells (MSCs) are a population of multilineage progenitor cells with the ability to differentiate into multiple mesenchymal lineages such as chondrocytes, osteoblasts, or adipocytes [1,2]. The initial isolation of MSCs was from the bone marrow (BM) based on plastic adherence of the cells as opposed BM hematopoieitic cells which can be cultured in suspension [3]. Increasingly, MSCs have been reported to be isolated from a number of other organs in the adult [4-7] and fetal-stage tissue [8-13]. Due to the difficulty in comparing the various methods used to isolate BM and tissue MSCs, a recent movement to define these progenitor cells have proposed a minimal criteria for MSCs in terms of trilineage mesodermal differentiation capacity and expression of a specific panel of cell surface marker including being positive for CD73, CD90, and CD105; and negative for hematopoietic markers such as CD14 or CD11b, CD34, CD45, and CD19 or CD79a [14]. The ease of isolation of MSCs along with reports of differentiation into extra-mesodermal cell types has made MSCs a popular choice for cell therapy for pre-clinical and clinical trials of a variety of diseases [15,16].

## 2. Immunomodulatory Properties of Adult and Fetal-stage MSCs

One important reason for the abundant number of clinical studies using adult BMMSCs is the immunomodulatory properties of these cells [17-20]. As with organ transplantation, a critical issue in stem cell therapy is the rejection resulting from immune incompatibility between donor and recipient. BMMSCs' immunomodulatory properties appear to obviate this major obstacle for cell therapy [21]; moreover, these immunosuppressive effects allow for an even wider range of disease indications for these progenitor cells, including use for immune-related diseases [4,22-26]. BMMSCs appear to be poorly immunogenic [27], since they constitutively express low levels of major histocompatibility complex (MHC) class I molecules and no MHC class II molecules. Moreover, BMMSCs do not express co-stimulatory molecules such as CD40, CD80, or CD86 which are involved in the activation of T cell for transplant rejection [18,28,29]. Several studies show that differentiated and undifferentiated BMMSCs have suppressive effects on alloantigen- and mitogen-stimulated lymphocyte proliferation in *in vitro *studies using mixed lymphocyte reactions (MLR), with a concomitant reduction in the production of proinflammatory cytokines such as interferon-γ (IFN-γ) and tumor necrosis factor-α (TNF-α)[17,18,30]. Thus, the clinical indications for human BMMSCs are considerably wider than other human stem cells, ranging from cell replacement for degenerative diseases--common indications for stem cell therapy--as well as immune-related diseases including autoimmune diseases and transplantation rejection [4,22-26].

While the differentiation plasticity and immunomodulatory properties of adult BMMSCs have brought much excitement in terms of prevalent clinical use for these progenitor cells, the fact remains that these cells are very rare, with cell numbers and proliferative capacity further decreasing with age [31,32]. In addition, an invasive procedure in terms of BM aspiration is needed to obtain BMMSCs. Thus, investigators have worked to identify other abundant and easily attainable sources of MSCs for therapeutic use. While many other adult tissues appear to harbor MSCs as well [4], the problems of requiring invasive procedures to obtain these relatively rare cells remain. A number of labs have thus turned to using discarded post-partum fetal-stage tissue for isolation of progenitor cells, since fetal umbilical cord blood is known to be a good source for the hematopoietic stem cell, one type of highly used stem cells. Known to be important in mediating the fetomaternal tolerance of pregnancy, fetal-stage extra-embryonic tissues are easily accessible sources for isolation of cells since these tissues are discarded at birth, obviating ethical issues as well.

Fetal MSCs (F-MSCs) have been derived from a number of fetal tissues, including fetal liver and bone marrow [13]. Moreover, extra-embryonic structures of fetal origin are also good sources for MSCs since they are discarded after birth, and MSCs isolated from human umbilical cord blood (UCB)(hUCB-MSCs)[9,33], the Wharton's Jelly (hWJ-MSCs) of the umbilical cord itself [34], amniotic fluid (AF)(hAF-MSCs)[10,35], amnion (hA-MSCs)[36], and the placenta (hP-MSCs)[8,37-39] have been demonstrated. These increasing reports on the isolation of MSCs from all these fetal-stage tissues demonstrate that F-MSCs can be an abundant and viable source of MSCs.

In addition to multilineage differentiation capacity, F-MSCs have been demonstrated to harbor strong immunomodulatory effects as well. F-MSCs lack or exhibit very low expression of highly polymorphic MHC class I (HLA-A, HLA-B, and HLA-C); furthermore, they do not express surface MHC class II molecules (HLA-DP, HLA-DQ, and HLA-DR) nor co-stimulatory molecules, such as CD40, CD40 ligand, CD80, and CD86 [10,34,35,40-52]. F-MSCs not only fail to induce an allogeneic or xenogeneic immune response in MLR, but also strongly suppress lymphocyte proliferation induced by mitogens or alloantigens, often in a dose-dependent manner [53-57]. Based on these data, F-MSCs appear to be as least as non-immunogenic as BMMSCs, and in some reports, appear to be even more immunomodulatory than its adult counterpart [40]. This is supported by clinical experience in hematopoietic stem cell transplantation (HSCT), where lower incidences of immune-related consequences are consistently seen after UCB HSCT compared to BM HSCT [21].

Based on the accumulating data, it appears that both adult-source MSCs--most prominently BMMSCS--and F-MSCs are good candidates for cell therapy for immune-related diseases in addition to degenerative diseases. In general, the overwhelming majority of the data is based on studies with BMMSCs rather than F-MSCs, since F-MSCs are newer sources of MSCs and have been studied only in the past few years. This review focuses on evidence for the immunomodulation of adult BMMSCs and F-MSCs, properties which appear increasingly relevant for clinical use. Interactions of BMMSCs and F-MSCs with subpopulations of leukocytes, and mechanisms of actions--when investigated--are reviewed.

## 3. Interactions of human BMMSCs and F-MSCs with leukocyte subpopulations

### 3.1 Interactions with T Lymphocytes

Currently, the interactions of MSCs with T lymphocytes are the best studied. Many reports have shown that BMMSCs affect several properties of T cells, most prominently efficiently suppressing activated CD4^+ ^T helper cell and CD8^+ ^cytotoxic T cell (CTL) proliferation [19,29,58-60]. Activated T cells are arrested by BMMSCs in the G0/G1 phase of the cell cycle [61], but apoptosis is not induced [19,29]. Besides their ability to impair the proliferation of activated T cells, BMMSCs can prolong the survival of unstimulated T cells by rescuing the lymphocytes from activation-induced cell death by down-regulation of Fas receptor and Fas ligand on T cells and inhibition of endogenous proteases involved in cell death [62]. Further studies have shown that BMMSCs reduce IFN-γ production by CD4^+ ^Th1 cells and interleukin (IL)-17 release by CD4^+ ^Th17 lymphocytes, whereas IL-4 secretion by CD4^+ ^Th2 cells is augmented [58,63-66]. The cytolytic potential of CTLs can also be efficiently impaired by BMMSCs [67]. Recently, several studies investigated the impact of BMMSCs on T regulatory lymphocytes (Tregs), a population of CD4^+ ^CD25^high ^cells which play an important role in the induction of peripheral tolerance and the inhibition of proinflammatory immune responses [68-70]. Many studies have shown that BMMSCs cultured with stimulated peripheral blood mononuclear cells (PBMCs) can induce the expansion of functional CD4^+ ^CD25^high ^Foxp3^+ ^Tregs [58,66,67,71-75]. A number of mechanisms have been suggested--both cell-contact dependent and independent mechanisms--but there is no clear consensus as of yet; for example, transforming growth factor-β (TGF-β) has been cited as being involved in one study [71] but not in another study [72]. This discrepancy may be due to the subtle phenotypic variations induced in BMMSCs by the many methods available for isolation of these adult stem cells.

Several studies have attempted to delineate which specific molecules are involved in the immunomodulatory effect of BMMSCs on T-cell proliferation and effector functions. In the human system, the effects of BMMSCs on T cells are mainly mediated through cell-contact independent processes, implicating the importance of secreted factors [76]. These molecules include IL-1β [77], TGF-β1 [19,71,77], hepatocyte growth factor (HGF)[19], prostaglandin E_2 _(PGE_2_)[58,71,78,79], indoleamine 2,3-dioxygenase (IDO)[59,79-81], heme oxygenase-1 (HO-1)[82], leukemia inhibitory factor (LIF)[83], insulin-like growth factor (IGF)[84], soluble human leukocyte antigen G5 (sHLA-G5)[74,85], galectin [86,87], and Jagged-1 [88]. Most of the inhibitory soluble factors are not constitutively secreted, but can be induced by the interaction between activated effector cells and BMMSCs (Table 1).

**Table 1:** Human BMMSC-Derived Immunoregulatory Soluble Factors

Leukocyte	Effects	Soluble Factors	References
T cells	Inhibition of T-cell proliferation, cytokine secretion and cytotoxicity	IL-1β	[77]
		
		TGF-β1	[19,71,77]
		
		HGF	[19]
		
		PGE_2_	[58,71,78,79]
		
		IDO	[59,79-81]
		
		HO-1	[82]
		
		LIF	[83]
		
		IGF	[84]
		
		HLA-G5/ other HLA-G	[74,83]
		
		Galectin-1	[87]
	
	Apoptosis of activated T-cells	IDO	[81]
	
	Generation of CD4^+ ^CD25^high ^Foxp3^+ ^Tregs	HLA-G5	[74]
		
		CCL1 (I-309)	[75]
		
		LIF	[83]

DCs	Inhibition of DC maturation	M-CSF	[98]

NKs	Inhibition of NK cell proliferation, cytokine secretion and cytotoxicity	TGF-β	[118]
		
		IDO	[59,119]
		
		HLA-G5	[74]
		
		PGE_2_	[59,118,119]

Abbreviations: DCs, dendritic cells; NKs, natural killer lymphocytes; Tregs, T regulatory lymphocytes; IL-1β, interleukin-1β; TGF-β1, transforming growth factor-β1; HGF, hepatocyte growth factor; PGE_2_, prostaglandin E_2_; IDO, indoleamine 2,3-dioxygenase; HO-1, heme oxygenase-1; LIF, leukemia inhibitory factor; IGF, insulin-like growth factor; HLA-G5, human leukocyte antigen G5; CCL1, CC chemokine ligand 1; M-CSF, macrophage-colony-stimulating factor.

**Figure 1:**
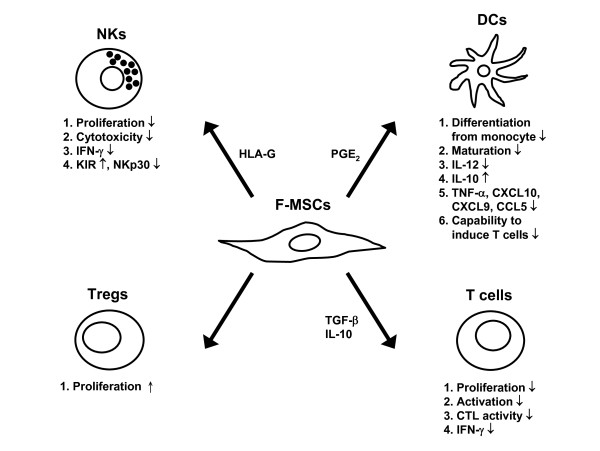
**Immunomodulatory effects of F-MSCs on different immune cells**. F-MSCs inhibit proliferation, cytokine secretion, and cytotoxic potential of NKs and CTLs. They also impair maturation, cytokine production, and T-cell stimulatory capacity of DCs. Moreover, F-MSCs inhibit the proliferation and cytokine secretion of T cells and promote the expansion of Tregs.

F-MSCs also have been reported to have strong inhibitory effects on T lymphocytes. hWJ-MSCs display potent immunosuppressive properties on T cell activation in an antigen-independent manner [51], and can also suppress the proliferation of mitogen-stimulated rat splenocytes (xenograft model) or human PBMCs (allogeneic transplant model) in allogeneic MLR *in vitro *[51]. Furthermore, CD14^+ ^monocytes promote the immunosuppressive effect of hWJ-MSCs probably via the IL-1β-PGE_2 _axis. The inflammatory cytokine IL-1β produced by hPBMCs upon activation upregulates the expression of cyclooxygenase-2 (COX-2) and the production of PGE_2 _by hWJ-MSCs [89-92]. hP-MSCs can also suppress the proliferation of allogeneic T cells [40,53,93]. These effects of hP-MSCs may involve the secretion of soluble factors TGF-β and IL-10 [40,94]. Both hP-MSCs and hUCB-MSCs have been shown to increase the proportion of Tregs, which contributes to the suppression of T cell proliferation [40,42](Figure 1).

Interestingly, a number of reports have demonstrated that in *in vitro *systems, pretreatment of BMMSCs and F-MSCs with the pro-inflammatory cytokine IFN-γ actually enhances their immunomodulation rather than decreases it [40,59,95]. Some investigators have postulated that this may explain the *in vivo *ability of MSCs to be effective against very inflammatory diseases such as graft-versus-host-disease (GVHD), in which the production of such activating cytokines as IFN-γ by T and natural killer lymphocytes (NKs) may actually promote MSC immunomodulation, subsequently suppressing the proliferation of CD4^+^, CD8^+ ^T cells, and NKs themselves [59]. While this has not been proven in animal studies, pre-clinical and clinical data continues to reveal therapeutic efficacy after MSC administration, giving indirect evidence for this hypothesis. Interestingly, while IFN-γ pre-treatment of adult BMMSCs results in induction of IDO, a strong immunosuppressive enzyme [59,80], MHC II molecules--which can elicit inflammatory responses [96]--are induced as well [12,40], but this does not appear to change the immunomodulatory effects of BMMSCs. It would be critical to elucidate this paradox to better understand why these progenitors possess such strong immunomodulatory properties inherently.

### 3.2 Interactions with Dendritic Cells (DCs)

DCs are derived from monocytes and are potent antigen-presenting cells (APCs) that act by internalizing, shuttling, and presenting antigens to naïve T-cells, which then leads to T-cell activation. These key regulators of immunity display an extraordinary capacity to induce T cell responses and secrete a variety of cytokines; the differentiation status of DCs can influence whether its target lymphocyte--often T cells--will mount an effector versus a more immunomodulatory response [97]. As such, studies have shown that BMMSCs inhibit the immunostimulatory capacity of DCs, supporting the development of a more tolerogenic population of DCs [93,98,99]. BMMSCs markedly impair PBMCs differentiation into DCs and inhibit endocytosis and the production of IL-12 by DCs. In the presence of BMMSCs, the differentiation of CD14^+ ^monocytes into DCs is impaired, and the monocytes retain high expression of CD14^+^--a marker of immaturity for DCs--without the upregulation of CD1a, HLA-DR, or co-stimulatory molecules which prevent the DCs to efficiently induce T cell effector responses [98]. In addition, BMMSCs also efficiently suppress the T cell-activating functions of DCs, including stimulation of T-cell proliferation, reduction of naïve CD4^+ ^T lymphocytes polarizing into proinflammatory Th1 cells, and promotion of Th2 responses. BMMSCs can decrease TNF-α secretion by DCs, which then leads to a reduced number of IFN-γ-producing Th1 cells. APCs generated in the presence of BMMSCs express low levels of IL-12, TNF-α, and MHC class II and high levels of IL-1β and IL-10, regardless of CD86 expression [100]. BMMSCs also induce DCs to secrete IL-10, which favors IL-4-producing Th2 cells and Tregs [58]. Furthermore, BMMSCs impair the release of cytokines by activated DCs through PGE_2 _[58,99]. Both cell-to-cell contact and soluble factors such as IL-6 and macrophage-colony-stimulating factor (M-CSF) mediate the BMMSC-mediated inhibition of differentiation, cytokine production, and T-cell stimulatory capacity of DCs [98,101](Table 1).

Interestingly, there are studies which show that BMMSCs itself can function as non-professional APCs. It has been reported that IFN-γ-stimulated BMMSCs can present exogenous antigens through upregulation of MHC class II molecules, which then results in activation of CD4^+ ^T cells [28,102-104]. BMMSCs can also cross-present exogenous antigens to induce CD8^+ ^T cell proliferation [105,106]. A few studies have shown that BMMSCs--similar to DCs--express high levels of toll-like receptors (TLRs), including TLR1, TLR3, TLR4, and TLR5. TLRs are receptors primarily expressed on APCs which recognize conserved pathogen-derived components. Triggering of TLR3, which binds double-stranded RNA, and TLR4, which binds lipopolysaccharide (LPS) and innate self antigens, on BMMSCs has been reported to suppress the immunomodulation of these cells through Notch ⁄ Jagged1 signaling, leading to production of pro-inflammatory mediators such as IL-1β, IL-6, and IL-8 [88,107-109]. However, another report showed that triggering of TLR on BMMSCs actually induces immunosuppression, which leads to the production of immunosuppressive kynurenines induced by IDO1. IDO1 can be induced by TLR3 and TLR4 signaling and this involves the activation of protein kinase R (PKR), an autocrine IFN-β signaling loop, and the activation of signal transducer and activator of transcription 1 (STAT1)/interferon regulatory factor 1 (IRF-1)[110]. These conflicting data regarding BMMSCs suppressing DC maturation and BMMSCs itself being an APC eliciting pro-inflammatory responses will require more research for clarification. One possible reason for these discrepant findings is that there is much heterogeneity between BMMSCs isolated from laboratory to laboratory. While the recent consensus of cell surface profile and tri-lineage mesodermal differentiation requirement has been extremely helpful to unify BMMSC phenotype [14], there still may exist epigenetic differences due to organ of origin and donor age, just to name a few variables. Moreover, the immunomodulatory properties of MSCs from different organs have not been much investigated, and one comparative study suggests that the MSC niche is unique in each tissue, which can contributes to functional differences [111]. Thus, it appears that studying the immunomodulatory behavior of MSCs derived from different origins would be important, and the accumulation of such data will help to shed more light and clarity on discrepant findings of this field.

Some studies have suggested that F-MSCs are poor APCs due to their low or limited expression of MHC class II and co-stimulatory molecules even after IFN-γ stimulation [40,112]. Recent studies have also investigated whether F-MSCs modulate DC phenotype and function. hAMSCs exert immunomodulatory effects on APCs, as demonstrated by their capacity to block maturation of monocytes into DCs [112]. They can prevent the expression of the DC lineage-specific marker CD1a and reduce the expression of HLA-DR, CD80, and CD83. This block in the monocyte-DC maturation process also results in impaired allostimulatory ability of these cells on allogeneic T cells [113,114]. Remarkably, hUCB-MSCs modulate DCs in a different way. hUCB-MSCs suppress the function of mature DCs by driving DCs to an intermediate maturation state and boosting IL-12 production by mature DCs [115]. These inhibitory mechanisms involve both cell-contact dependent as well as secretion of soluble factors [50](Figure 1).

### 3.3 Interactions with Natural Killer Lymphocytes (NKs)

NKs are key players of the innate immune system and are important in targeting virus-infected cells and tumor cells. NKs are highly cytotoxic and secrete large amounts of proinflammatory cytokines such as TNF-α and IFN-γ [116,117]. Part of the innate immune system, these cytotoxic lymphocytes are triggered to recognize and respond to MHC molecules signifying "self" versus "non-self", rather than specific antigens which T and B lymphocytes of the adaptive immune system recognize. A few studies have shown that BMMSCs are able to suppress the proliferation and cytokine production of NKs [58,67,118]. The inhibition requires both cell-to-cell contact and soluble factors such as PGE_2 _and TGF-β [59,118]. BMMSCs can also modulate the cytotoxicity of NKs, reducing the levels of NK-secreted cytokines such as IFN-γ, IL-10, and TNF-α and this phenomenon also requires cell-cell contact [118,119]. However, stimulated NKs can efficiently lyse autologous and allogeneic BMMSCs [118,120,121]. The activating NK receptors NKp30, NKG2D, and DNAM-1 were involved in NK-mediated cytotoxicity against BMMSCs. IFN-γ-stimulated BMMSCs, on the other hand, were less susceptible to NK cell lysis as a consequence of the up-regulation of MHC class I molecules at the MSC surface [121]. Moreover, the secretion of soluble HLA-G (sHLA-G) by BMMSCs plays an important role in the inhibition of NK cytotoxicity and IFN-γ release [74]. First identified in choriocarcinoma and migratory trophoblasts, HLA-G (non-classical MHC I molecule) is thought to confer for the fetus a protective effect against the maternal immune system, including directly suppressing maternal NK cytotoxicity [122]. HLA-G can exist in several forms, with the best characterized being the complete transmembrane form (HLA-G1)--the predominant *in vivo *form--and one of the three soluble, truncated forms (HLA-G5 or sHLA-G)[123]. Unlike most MHC I molecules, HLA-G has very low polymorphism and its expression in the adult is highly restricted; however, in certain pathologic states including cancer and inflammatory diseases, expression can be induced [124]. The receptors for HLA-G include ILT-2, ILT-4, and CD94 and these receptors can be found on a number of leukocytes, most prominently being NKs [125](Table 1).

F-MSCs can express surface molecule HLA-G, indicating potential tolerance-inducing properties [51,126]. We found that hP-MSCs are more resistant to stimulated-NK cytotoxicity than BMMSCs; moreover, hP-MSCs demonstrate enhanced suppressive effects towards NK in the presence of IFN-γ, and this is partially mediated through surface expression of HLA-G on hP-MSCs but not adult BMMSCs [127]. The placenta is known to have unique immunomodulatory interactions with maternal uterine NKs, which also have a different effector profile than peripheral blood NKs [128,129]. Thus, interactions of F-MSCs with NKs may be quite different than that found with BMMSCs, since NKs are one of the most important and predominant lymphocyte populations found during pregnancy. While such data is still scarce, research on F-MSCs interactions with this population of innate lymphocytes should yield interesting data, and perhaps even shed light on maternal-fetal immune mechanisms (Figure 1).

## 4. Clinical applications of adult and F-MSCs for GVHD

The majority of data on the immunomodulation of MSCs are *in vitro *in nature, however, a number of studies have been *in vivo*. One of the potentially lethal consequences after allogeneic HSCT is GVHD in which recipient cytotoxic T cells attack donor tissue, resulting in an immune-related complication which is associated with high morbidity and mortality [130]. Animal models of GVHD are one of the most commonly used disease models to validate BMMSC immune function *in vivo*, and these studies have demonstrated that BMMSCs do remain immunomodulatory *in vivo *[131-134]. Recent data has shown that the combination of BMMSCs and immunosuppressive drugs can prolong organ allograft survival [135,136]. Because of the profound immunomodulatory effect of BMMSCs shown *in vitro *and *in vivo*, co-transplantation of *ex vivo*-expanded BMMSCs with HSCs for GVHD has been recommended [22,26,137-145]. In addition, cytokines released by BMMSCs may promote homing or proliferation of HSCs and enhance HSCs engraftment [146-151]. Thus, based on the accumulated *in vitro *and animal studies, a number of clinical trials have been started to evaluate the potential of BMMSCs for the treatment of GVHD [22,26,137,140,152].

While there are in general fewer studies using F-MSCs as a cell therapy source, some pre-clinical studies have been conducted. Several animal studies show the prolonged survival of hAMSCs with no evidence of immunological rejection after xenogeneic transplantation into immunocompetent animals including rats [56,153-155] and swine [56]. Moreover, hAF-MSCs appear to be relatively resistant to rejection by the recipient--even with allogenic cell transplantation--due to the expression of immunosuppressive factors such as CD59 (protectin) and HLA-G [156]. Co-transplantation of UCBHSCs along with F-MSCs can reduce potential GVHD in recipients [35], as well as enhance UCB cell engraftment and homing of CD34^+ ^HSCs [157,158]. Therefore, F-MSCs appear to be a promising source for stem cell therapy of GVHD and likely other immune-related diseases.

## 5. Conclusions

MSCs are multilineage progenitors which can be isolated from many adult organs as well as fetal-stage tissues. BMMSCs and F-MSCs have been reported to harbor strong immunomodulatory effects. While the data is still scarce regarding F-MSCs, several differences in the immune-suppressive properties between F-MSCs and adult BMMSCs have been found. Future investigations on the molecular mechanisms underlying the immunomodulatory properties of both F-MSCs and adult BMMSCs would be important since these differences may have functional relevance to therapeutic use of both sources of progenitor cells.

## Competing interests

The authors declare that they have no competing interests.

## Authors' contributions

All authors have read and approved the final manuscript.

## Acknowledgements and Funding

This work was supported by the National Science Council of Taiwan (grants NSC-99-3111-B-400-002, NSC 97-2320-B-400-005-MY3, and NSC-99-3112-B-400-006 to B.L.Y.; and NSC-99-3111-B-002-009 to M.L.Y.).
